# Necrological

**Published:** 1881-02

**Authors:** 


### NECROLOGICAL.

" Death is the crown of life:
Were death denied, poor man would live in vain."

Dr. Samuel T. Knight of this city, died suddenly at his residence, on the 20th ultimo. Dr. Knight was in his 64th year, and had been engaged in active practice here for many years. He was a polite unassuming gentleman, and greatly esteemed by his patients and associates. He was a member of the Society of Friends, and was buried in accordance with the customs of that order.

Dr. William H. Davis, an old and highly respected physician, and also a member of the Society of Friends in this city, was called to his home above, six days after Dr. Knight.

Dr. Levin S. Joynes, a distinguished Physician and Professor of Richmond, Va., has recently died in his 63d year of age.

